# Supplementary material for: Are carbon emissions trading and green financial instruments synergistic? -Comprehensive quantitative research based on content analysis
Source: PLoS One. 2024 Mar 7;19(3):e0298601. doi: 10.1371/journal.pone.0298601 (PMC10919601; doi:10.1371/journal.pone.0298601)
Supplement: S1 Data — (ZIP) [file pone.0298601.s001.zip › Data/地级市Z_F工作报告.zip!/╜¡╬≈/╙Ñ╠╢╩╨/└┤╫╘╣1⁄2╓┌║┼í╛┬φ┐╦╩2╛▌═°í┐_2013.docx]

各位代表：

　　现在，我代表市人民政府向大会作政府工作报告，请予审议，并请市政协委员和列席会议的同志提出意见。

　　一、2012年工作回顾

　　过去的一年，在省委、省政府和市委的正确领导下，全市上下按照“主攻项目，决战‘三区’，凸现特色，实现跨越”的总体要求，攻坚克难，扎实工作，实现了经济社会持续健康较快发展。预计完成生产总值482亿元，增长11.7%；财政总收入78.7亿元，增长10.8%；地方财政收入58.3亿元，增长50%；规模以上工业增加值280亿元，增长15%；全社会固定资产投资410亿元，增长22%；社会消费品零售总额115亿元，增长14.6%；城镇居民人均可支配收入19880元，增长13.5%；农民人均纯收入8600元，增长13%。

　　（一）重点产业快速发展。全市规模以上工业实现主营业务收入2400亿元，位居全省第三。鹰潭高新区成功升格为国家高新技术产业开发区。铜产业完成主营业务收入2130亿元，增长17.2%。江铜30万吨铜拆解项目一期竣工投产。鹰潭（贵溪）铜产业循环经济基地被评为全国循环经济工作先进单位。65个战略性新兴产业项目进展顺利，完成投资100亿元以上，我市荣获全省推进战略性新兴产业先进市称号。龙虎山成功创建国家“5A”级旅游景区，接待游客506.3万人次，增长36%；旅游直接收入2.13亿元，增长33%。全市接待国内外游客1100万人次，增长30%；旅游总收入76亿元，增长30%。物流产业实现营业收入130.5亿元，增长83.6%；上缴税收5亿元，增长62.2%。制定了文化休闲娱乐等服务业发展规划，举办了首届中国（鹰潭）中华赏石展暨黄蜡石文化博览会。鹰潭农商银行挂牌开业。

　　（二）城市建设步伐加快。实施重大城建项目104个，总投资248亿元。信江新区路网已经成型，鹰潭一中新校区投入使用，高铁北站、市行政服务中心、滨江景观工程等重点项目稳步推进，信江新区完成征地1.4万亩，净地交付1.2万亩，拆迁面积38.6万平方米。滨江公园二期、林荫东路综合改造等一批老城区改造项目顺利竣工，城市展示馆（市民服务中心）即将开馆，惠及3万人的百佳城、山背小区、立新巷等小街小巷综合改造项目全面完成，海关国检大楼、梅园迎宾馆、林荫西路贯通、四海路延伸、市人民医院片区整体改造等项目全面实施，北极阁重建项目顺利开工。“三城同创”有序推进。月湖新城开工项目12个，贵溪城区提升改造成效显著，余江县城东拓稳步推进，龙虎山上清古镇一期改造基本完成，“一江两岸、四区合一”城市发展格局初步形成。

　　（三）“三农”工作成效明显。粮食总产达67.8万吨，实现“九连增”。鹰南贡米被评为国家级农业产业化龙头企业，全市农业产业化龙头企业达125家，实现销售收入52亿元，增长20%。242个自然村点的新农村建设和贵冶周边11个自然村环境整治任务全面完成，“三区三线”民居改造提升工作有序实施，“秀美乡村”创建活动不断深入。实施水利项目127个，完成中潢圩堤等五河治理应急除险和40座小型病险水库除险加固任务。完成造林绿化5.86万亩。阳际峰自然保护区成功晋级为国家级自然保护区。

　　（四）改革开放不断深化。围绕建设“四个鹰潭”、发展“1+6”产业、促进县域经济竞相发展、做强金融业、完善国资监管体系等，制定出台了一系列改革举措。开通了海铁联运国际运输通道和鹰潭至宁波“五定班列”。中国四冶与中国节能环保集团成功合作，步入发展快车道。百炼集团、绿地控股集团、浙商联盟等一批知名企业落户我市。全年实际利用外资1.68亿美元，增长15.9%；引进省外5000万元以上项目资金170亿元，增长18.5%；外贸出口7.1亿美元，增长16.5%。我市荣获全国外贸百强城市、国家外贸转型升级示范基地称号。

　　（五）改善民生取得实效。全面完成了省政府下达的89项民生指标，教育、社保、医疗、住房等民生支出达35.6亿元。全市新增城镇就业2.7万人，新增转移农村劳动力2.1万人，城镇就业率达96.5%。实现了城乡居民社会养老保险全覆盖，“新农合”参合人数79.4万人，参合率达96.3%。我市被国务院授予全国新型农村和城镇居民社会养老保险工作先进单位。发放城乡低保资金1.2亿元，城乡最低生活保障标准分别提高到350元和170元。为老年人、婴幼儿、慢性病人等重点人群提供免费健康体检33.7万人次。开工建设各类保障性住房6418套，民欣家园1640套廉租房交付使用，中心城区保障性住房覆盖率达23%，提前完成“十二五”规划目标。

　　（六）社会事业协调发展。编制了鹰潭市城区教育设施布局专项规划，全面启动了高教园区建设，校安工程竣工面积9.1万平方米，拆除D级危房11.2万平方米。市人民医院门诊医技楼、市中医院综合楼投入使用，国家基本药物制度实现行政村全覆盖。扎实推进了科技创新“六个一”工程，列入国家和省科技计划项目40项。文化体制改革基本完成。《七彩畲乡》荣获第四届全国少数民族文艺会演剧目金奖，创造了江西少数民族文化的新辉煌。我市连续17年荣获全国全民健身活动优秀组织奖。食品药品市场秩序日趋规范。44个社区标准化建设和社区服务管理网格化工作试点进展顺利。社会管理综合治理形势平稳。

　　统计、审计、监察、人事编制、法制、司法行政、人口计生、公路交通、安全生产、行政服务、方志档案、广播电视、新闻出版、民族宗教、外事侨务、涉台事务、气象服务、防灾减灾、质量监督、民兵预备役、国防动员、拥军优属、人民防空、妇女儿童、老龄、残疾人等工作取得了新的成绩。一年来，全市荣获省级以上荣誉73项。

　　各位代表，成绩和进步来之不易。这些成绩的取得，是省委、省政府和市委正确领导的结果，是市人大依法监督、市政协民主监督的结果，是全市人民锐意进取、顽强拼搏的结果。在此，我代表市人民政府，向全市广大干部群众，向各位人大代表、政协委员、各民主党派、工商联、无党派人士和社会团体，向驻鹰单位、人民解放军、武警官兵、公安民警和离退休老同志，向所有关心、支持、参与鹰潭经济社会发展的各界人士，表示衷心的感谢并致以崇高的敬意！

　　回顾一年的工作，我们深深体会到：

　　必须始终坚持发展目标不动摇。建设富裕、秀美、宜居、和谐鹰潭，是市委市政府贯彻省委省政府发展战略、深化市情认识作出的科学决策，是打造鄱阳湖生态经济区璀璨明珠的内在要求，是深入落实党的十八大精神、全面建成小康社会的有效举措。一年来，我们坚持在建设"四个鹰潭"中统一思想，在统一思想中加快“四个鹰潭”建设，实现了全市经济社会平稳较快发展。

　　必须始终坚持总体要求不动摇。“主攻项目，决战‘三区’，凸现特色，实现跨越”的总体要求，是鹰潭实现跨越发展的手段、载体、方向的有机统一。一年来，我们坚持把“主攻项目”作为主抓手，深入实施项目带动战略，围绕优势产业抓项目，依托独特资源抓项目，发挥区位优势抓项目，紧扣特色产品抓项目；把“决战‘三区’”作为主战场，全力将园区、城区、景区打造成经济社会发展的重要增长极；把“凸现特色”作为主方向，扎实做好铜产业、旅游业、物流业“三篇文章”，促进了经济社会持续健康发展。

　　必须始终坚持解放思想不动摇。实践发展永无止境，解放思想永无止境。一年来，我们坚持用开放的办法破解发展难题，用改革的举措理顺体制机制，不断推进思想观念创新、体制机制创新、管理方式创新，发展动能加速释放，发展活力不断增强。

　　必须始终坚持改善民生不动摇。民生连着民心，民心凝聚民力。一年来，我们坚持把保障改善民生作为政府的最大责任，在实际工作中，既考虑政府为群众做什么、更注重群众需要我们做什么，既关心群众现实利益、更关注群众切身感受，既重视个体民生问题、更重视群体民生问题，不断满足人民群众过上更好生活新期待，让改革发展成果更多更好地惠及广大人民群众。

　　必须始终坚持真抓实干不动摇。政府工作，贵在落实，重在实干。一年来，我们坚持“没有任何借口”的工作理念，把“少说多做看结果”作为检验工作的重要标准，注重统筹、服务、督查，突出抓重点、攻难点、出亮点，强化理解力、执行力、操作力，以求真务实的作风推动各项工作的全面落实。

　　在肯定成绩的同时，我们也清醒认识到，我市经济社会发展还存在不少困难和压力。一是受宏观调控政策影响，经济下行压力加大，产业发展面临困难。二是财政增速趋缓，财源培植难度加大。三是中心城区人口规模偏小，功能配套不够完善，城市承载力、辐射力、带动力不强。四是部分重点项目进度不快，征地拆迁相对滞后。五是教育特别是学前教育等民生工作与群众的需求和期盼存在差距，社会事业发展和社会管理创新的任务仍然艰巨。对此，我们将采取有力措施，切实加以解决。

　　二、2013年主要工作

　　2013年是贯彻落实党的十八大精神的开局之年，是为全面建成小康社会奠定坚实基础的重要一年，是建市30周年的喜庆之年。做好今年的工作，对我市在全省区域发展格局中更有作为、更有位置、更有形象，具有十分重要的意义。

　　2013年政府工作的总体要求是：高举中国特色社会主义伟大旗帜，以邓小平理论、“三个代表”重要思想和科学发展观为指导，全面贯彻落实党的十八大精神，按照“主攻项目，决战‘三区’，凸现特色，实现跨越”的总体要求，更加注重产业培育，更加注重城市品质，更加注重改革开放，更加注重改善民生，更加注重城乡统筹，更加注重生态文明，开拓创新，扎实工作，加快建设富裕、秀美、宜居、和谐鹰潭，以优异成绩迎接建市30周年。

　　2013年全市经济社会发展的主要预期目标是：生产总值560亿元，增长11%。财政总收入90.5亿元，增长15%；其中，地方财政收入63.6亿元，增长9.2%。全社会固定资产投资500亿元，增长21%。社会消费品零售总额134亿元，增长16%。城镇居民人均可支配收入22200元，增长12%。农民人均纯收入9600元，增长12%。居民消费价格总水平涨幅控制在3.5%以内。人口自然增长率控制在7.6‰以内。万元生产总值能耗下降3.4%，化学需氧量、二氧化硫、氨氮、氮氧化物排放量控制在省下达目标以内。

　　围绕上述目标，重点抓好以下七个方面的工作：

　　（一）突出项目建设，力促跨越发展。

　　深入实施项目带动战略，以30个重点产业项目、重大城建项目、重要民生项目为抓手，转变发展方式，优化经济结构，以大项目促进大投入、以大投入实现大发展。

　　1.全力以赴推进项目。全年安排重大建设项目277个，总投资720亿元，当年计划完成投资300亿元。加快推进重大项目建设，着力实施好胜华集团电线电缆、上诠集团光纤、海尚科技纳米锌等10大重点产业项目，着力实施好信江新区中央商务区、老城区道路管网改造、城市绿化景观等10大重大城建项目，着力实施好保障性安居工程、社区标准化和便民中心、污水处理厂等10大重要民生项目，完成投资200亿元。全力服务中央、省属和过境项目建设，积极配合杭长客运专线鹰潭段、江西成品油管道二期工程鹰潭段、沪昆高速龙虎山服务区等14个重大项目建设，完成投资100亿元。

　　2.全力以赴争取项目。研究政策，用足政策，紧盯国家投资取向，做好项目争取工作。完善全市、部门（系统）项目库，精心包装推介一批有发展潜力、能带动全局的项目。创新“三争”机制，整合各方资源，加强沟通对接，全力争取国家级铜产业基地、绿色水工产业基地、循环经济和资源综合利用、产业振兴和中小企业改造等44个重大项目，力争项目早审批、资金早到位、政策早实施。

　　3.全力以赴服务项目。强化机制保障，全面落实“一个项目、一名领导、一支队伍、一套措施、一抓到底”的工作机制，着力提高项目落地率、开工率、竣工投产率。强化要素保障，着力解决项目融资、用工、用地等要素制约。强化环境保障，本着“政策跟着项目走、服务贴着企业做”的原则，大力推进网上审批和公共资源网上交易，努力为企业落户、项目建设、产业发展提供优质高效的服务。

　　（二）做强工业经济，壮大综合实力。

　　围绕“决战工业5000亿，实现鹰潭新跨越”的目标，坚持做大总量与调整结构并举、提高质量与优化效益并重，深入实施“1+6”产业项目竞赛年活动，确保规模以上工业增加值达320亿元，主营业务收入达2700亿元。

　　1.增强产业竞争力。强攻主导产业。深化与江铜集团一体化合作，全面落实支持服务江铜发展若干政策措施，充分发挥海铁联运和“五定班列”的优势，推进铜拆解加工区二期工程建设，加快江铜30万吨铜拆解项目建设，形成政策洼地效应，实现铜拆解量10万吨。大力引进电线电缆、水暖卫浴等铜精深加工企业，争取年内动工建设铜期货交割仓库，加快培育铜现货交易市场，建立铜科技研发平台，投入使用国家铜及铜产品质量监督检验中心，大力发展铜文化创意产业，努力打造世界级铜基新材料产业基地，确保全年实现铜产业主营业务收入2400亿元。做大特色产业。加快三川水工产业园二期建设，完成12万平方米厂房建设和水表车间搬迁，着力引进一批水工企业，打造全国最大的水工产业基地。加快节能照明产业园建设，大力发展节能照明电器及其配套产业，全力打造“中国灯谷”。加快汽摩配产业园、硫磷化工产业园等省级产业基地建设，推动万宝至马达二期、百炼新型氟材料等项目投产见效。加快鹰潭国际眼镜城建设，实现从单一的眼镜销售向眼镜生产基地转型，做响“眼镜之乡”品牌。加快雕刻创业园、雕刻一条街建设，大力发展雕刻产业。培育新兴产业。加快大健康产业园建设，依托康恩贝、天施康药业、华宝集团等龙头企业，建设集生物医药、食品工业、中药生态农业为一体的大健康产业集群，全力打造百亿大健康产业基地。加快金泰新能源、同人电子等新能源新材料项目建设，全力打造区域性新能源新材料示范基地。

　　2.夯实园区承载力。围绕打造“千亿园区、工业新城”，编制工业新城总体规划、产业布局规划和功能区规划，加快白露科技园、龙岗产业园建设，积极搭建招商共享平台、高新企业孵化平台、小微企业创业平台、科技金融服务平台，建设大学科技园、总部商贸大楼，完善生活和服务设施配套，确保鹰潭高新区实现主营业务收入420亿元。围绕扩园升级，完善基础设施，促进产业集聚，确保贵溪、余江工业园区完成主营业务收入580亿元。

　　3.提升企业创新力。着力增强创新驱动发展新动力，积极搭建科技创新平台，加强产品创新、品牌创新、产业组织创新、商业模式创新。深入实施科技创新“136”工程，鼓励支持企业建设技术研发机构，开展产学研合作，引导创新要素向企业集聚，加快科研成果转化，壮大龙头企业，做优主导产品，掌握核心技术，力争申请专利320件以上，申报高新技术企业4家以上，实现高新技术产业产值260亿元，增长20%。

　　（三）加快城市建设，做靓“一江两岸”。

　　坚持以“三城同创”为载体，以规划设计为龙头，以信江新区建设为中心，以完善老城功能配套为重点，加快构建“一江两岸、四区合一”的大框架城市格局，以崭新的城市形象迎接建市30周年。

　　1.精心做好规划设计。完成信江新区北区控规编制及南区控规修编、鹰东组团和老城西区控规修编、南站北区控规编制，进一步优化中心城区各区块功能定位。扎实做好信江新区中央商务区、鹰西湿地公园、信江污水处理厂等重点项目设计，抓好中心城区主干道店招、广告等专项设计，提升中心城区主要通道、重要节点、重点建筑的设计水平，打造精品亮点，彰显城市魅力。

　　2.加快建设信江新区。高标准完成龙虎山大道、信江大道、滨江大道、余信贵快速通道等主干道油化、亮化、绿化、美化工程，构建循环畅通的信江新区路网体系。建成市民广场、滨江景观工程，加快推进市行政服务中心、高铁站前广场、信江步行街、中央生态公园、沿江公园、虎岭调蓄池公园、刘家调蓄池公园等重点项目建设，以鹰潭大厦、果喜大厦、鹰潭农商银行、上饶银行等项目为重点，全面推进信江新区中央商务区建设，启动余信贵大桥、信江污水处理厂建设。加快实施一批房地产项目，做旺信江新区人气。加大依法征地拆迁力度，满足项目用地需求。

　　3.完善老城功能配套。全面启动仪表厂、二二三队、中铁二十四局片区棚户区改造，加快鹰西片区、铁路片区、防腐厂片区棚户区和桃李村、梅园詹家、白马李家等“城中村”改造步伐。完成赵家弄、莲花路、沙塘石等7个片区小街小巷综合改造，贯通林荫西路、四海路、天洁东路，实施城西片区排水管网改造和生活垃圾焚烧发电项目。建成梅园迎宾馆、天裕豪生酒店、北极阁重建项目，完成鹰西、白马菜场建设。加快建设鹰西湿地公园、枫山植物园、白鹭公园，改造提升东湖公园、月湖岩公园、月牙湖公园，兴建一批街头小绿地、小广场、停车场，确保成功创建国家园林城市。加快“数字城管”建设，有序推进城市管理体制机制改革，不断提高城市网格化、精细化管理水平。加大月湖新城建设力度，加快贵溪东城区城市综合体、余江新城区开发建设，进一步拉大城市框架，完善城市功能。

　　（四）大力发展现代服务业，繁荣城市经济。

　　繁荣壮大以旅游和物流为主导、以文化休闲娱乐为重点的现代服务业，打响“玩在鹰潭、乐在鹰潭”品牌，确保服务业增加值占生产总值比重达28%。

　　1.决战景区，做大旅游产业。拉开大框架，谋划大项目，拓展大市场，把龙虎山打造成集自然观光、道教体验、养生度假、休闲娱乐为一体的旅游目的地。深入实施龙虎山片区旅游发展概念性规划，积极推动龙虎山旅游风情小镇、上清道源古镇、天禄旅游集散中心等功能区建设，构建“一廊三区、四核多片”发展格局。重点推进景区路网、水网、电网建设，加快上清至饶桥旅游公路、水寨宋庄、天元度假区、圣井山综合开发等重点项目建设，改造升级龙虎山竹筏码头，提升景区承载功能。进一步完善龙虎山旅游文化发展集团有限公司体制机制，推进龙虎山旅游向品质化经营、精细化管理转型。力争全市接待国内外游客突破1400万人次，旅游总收入突破100亿元；其中，龙虎山景区接待游客690万人次，旅游直接收入2.66亿元。

　　2.依托区位，做强物流产业。围绕建设鄱阳湖生态经济区物流中心城市和全国区域性物流节点城市，加快建设现代物流园、鹰西商贸物流园，全力推进机电五金商贸城、手拉手国际汽车城、建材家居城、粮食市场、农副产品市场、食盐物流储备配送中心等项目建设。加快建设货运配载中心、转运中心和物流信息平台，引导货运企业转型升级。力争物流产业实现营业收入180亿元、税收6.5亿元。

　　3.彰显特色，做旺文化休闲娱乐等服务业。深入挖掘道文化等传统文化内核，抓好龙虎山文化艺术创意园、大道龙虎山建设，加快文化创意产业园、滨江休闲风情带、特色文化商业街、城市特色旅游区等项目建设步伐，积极打造以鹰潭黄玉为主导的华东地区玉石交易集散中心，努力把鹰潭建设成为区域性文化休闲娱乐目的地。争取银行、证券、保险等金融机构来鹰设立分支机构，支持发展村镇银行、小额贷款公司和融资性担保公司，加强民间金融的监管。全力支持鹰潭农商银行做大做强，力争年内累计信贷投放超40亿元、税收超5000万元。善待和支持小微企业发展，健全支持实体经济发展的多层次融资体系。做好企业上市工作。

　　（五）统筹城乡发展，做好“三农”工作。

　　加快城乡统筹是解决“三农”问题、缩小城乡差距的根本途径。以全省统筹城乡综合配套改革试点为抓手，坚持以城带乡、以工促农，加大强农惠农富农政策力度，增强农村发展活力，促进城乡共同繁荣。

　　1.以创建白鹤湖国家级现代农业综合示范区为龙头，大力发展现代农业。按照“一年打基础、三年出形象、五年大发展”的要求，扎实推进规划面积145平方公里的白鹤湖国家级现代农业综合示范区建设，全力打造“一带一核五区”。积极整合资源，加快雷溪万亩现代农业科技示范区建设。深入实施粮食高产创建工程，建设高标准农田4万亩，确保粮食总产稳定在66万吨以上。创新农业生产经营体制机制，着力发展多种形式的新型农民合作组织和多元服务主体，培育一批年产值超5亿元的农业产业化龙头企业，力争市级以上农业产业化龙头企业销售收入突破60亿元。加强绿色生产，从源头上确保农产品质量安全。

　　2.以打造“秀美乡村”为重点，大力完善农村基础设施。深入推进“秀美乡村”创建活动，完成200个以上自然村点的新农村建设任务，打造一批精品示范区。全面完成“三区三线”民居改造提升工作。深入实施信江新区港口段、余江城区等城市防洪工程，完成龙虎山景区防洪等一批中小河流治理以及70座小（2）型水库除险加固工程，兴建一批千吨万人集中供水项目，解决5.7万农村人口饮水安全问题。做好花桥大型水库项目前期工作。改造建设农村公路260公里。

　　3.以“森林城乡、绿色鹰潭”创建活动为载体，大力优化生态环境。把生态文明建设放在突出地位，深入实施“森林城乡、绿色鹰潭”建设，完成造林绿化5.3万亩，确保森林覆盖率57.6%以上、城市建成区绿化覆盖率42%以上。加快实施贵冶周边第二批10个自然村环境整治工作。深入推进农村清洁工程，逐步推广户分类、村收集、乡集中、县运输、市处理的农村垃圾无害化处理模式。加大农业面源污染治理力度，发展农业循环经济。

　　（六）深化改革开放，激发发展活力。

　　改革为发展提供动力，开放为跨越注入活力。坚定不移实施大开放主战略,在扩大开放中赢得发展机遇；积极稳妥推进重点领域和关键环节改革，在深化改革中拓宽发展空间。

　　1.招大引强，扩大开放。牢固树立“让大利、招大商、大发展”的理念，推动招商引资朝着优化结构、拓展深度、提高效益方向转变，确保全市实际利用外资1.85亿美元，引进省外5000万元以上项目资金195亿元。突出产业招商，重点围绕“1+6”产业和旅游、物流、农业产业化等，大力引进龙头型、潜力型、绩优型、补链型企业，不断提高引进项目的投资总量、科技含量、纳税数量和就业容量。注重靠大联强，深化与中国节能环保集团等央企的战略合作，为发展注入强劲动力。夯实开放平台，建成海关国检大楼，开通鹰潭电子口岸，争取年内开关开检，形成关、检、贸一体化大通关格局。扩大外贸出口，确保全年实现外贸出口7.38亿美元。

　　2.创新机制，深化改革。继续深化医药卫生体制改革，加快县级公立医院综合改革试点步伐，鼓励社会资本办医。完善国库集中支付制度，全面推行公务卡改革，规范部门预算编制，推进政府采购改革。注重税负率、征收率、可用率、能耗产出率，强化财税征管。实施园区再造工程，加快构建土地管理新格局。深入推进水利综合改革试点工作。稳步推进国有企业厂办大集体和国有林场改革。

　　（七）保障改善民生，促进社会和谐。

　　保障民生是政府最大的责任，改善民生是政府最大的政绩。始终把人民群众摆在心中最高位置，提高居民收入，加强社会建设，创新社会管理，使发展成果更多、更公平惠及全体人民，努力开创和谐社会人人共享的生动局面。

　　1.深入实施民生工程。全面完成省政府下达的各项民生指标。实施就业优先战略和更加积极的就业政策，确保新增城镇就业2万人，新增转移农村劳动力1.3万人。完善养老保险“三张网”，推动养老保险由制度全覆盖向人群全覆盖迈进。完善最低生活保障与物价上涨挂钩联动机制，提高最低生活保障标准和补助水平。扩大大病救治范围，将新农合筹资标准提高到340元，参合率保持在95%以上。开工建设公共租赁住房4008套，完成城市棚户区改造908套、垦区林区危旧房改造2635套，加快构建“三房合一、租售并举”的保障性住房体系。

　　2.协调发展社会事业。加大教育资源整合力度，努力办好人民满意教育。

　　在全面提升义务教育均衡发展水平的基础上，重点抓好学前教育和职业、高等教育。以壮士断臂的决心办好学前教育，落实学前教育三年行动计划，加大公办幼儿园建设力度，县（市、区）建若干个示范幼儿园，每个乡镇至少建一所标准化幼儿园，充分发挥公办幼儿园的示范作用；加大民办幼儿园扶持力度，对新建、改建、扩建民办幼儿园给予用地优惠，提供三年贴息补助，对达标创优幼儿园实行财政奖励，对更换标准校车给予财政补助。通过三年努力，整体提升学前教育办学水平，让孩子舒心、家长放心、社会满意。完成市人民医院信江院区、市精神病医院主体工程建设。加强以全科医生为重点的基层医疗卫生队伍建设，加大儿童先天性心脏病、白血病、尿毒症血透等21种重大疾病的免费治疗和救助力度。推进省级食品药品安全示范区建设，提升食品药品安全保障水平。深入开展全民健身活动，办好市第七届运动会和首届体育文化节。做好人口计生工作，稳定低生育水平。发行《龙虎山》特种邮票。加强国防后备力量建设，深入开展“双拥”共建活动，巩固发展军政军民团结。充分发挥统计、审计、物价、质监、气象、防灾减灾、民族宗教、外事侨务、涉台事务、广播电视、新闻出版、档案方志、妇女儿童、老龄、残疾人等工作在构建和谐社会中的促进作用。

　　3.加强创新社会管理。以44个社区标准化建设为载体，全面推进社区服务管理信息化、网格化建设，夯实社会管理服务基层基础。健全市、县、乡、村（社区）四级矛盾纠纷排查化解网络，完善人民调解、行政调解、司法调解“三调联动”机制，切实解决影响社会和谐稳定的基础性、源头性、根本性问题。推进视频监控系统联网建设及运用，完善社会公共安全体系。强化安全生产，突出抓好重点领域、重点行业、重要场所的安全监管，确保生产安全、交通安全、消防安全、校园安全，坚决遏制重特大事故的发生，切实增强公众安全感。健全重大决策社会稳定风险评估机制和应急管理机制，提高突发公共事件防范处置能力。

　　各位代表，事业的发展、人民的期盼，要求我们进一步加强政府自身建设。我们要按照“干部清正、政府清廉、政治清明”的要求，改进作风，为民做事。认真执行改进工作作风、密切联系群众“八项规定”，坚持问政于民、问需于民、问计于民，多到困难和矛盾集中、群众意见多的地方去，多干群众期盼、百姓急需、人民受益的事，使政府工作更好地体现民意、顺应民心。强化执行，务实成事。坚持立说立行，做到执行上“没有任何借口”、操作上“少说多做看结果”；坚持勇于担当，做到矛盾面前不回避，困难面前不退缩，责任面前不推诿；坚持一抓到底，明确目标进度，突出过程监管，严格督查问责，力促政府工作快节奏、高效率。依法行政，公正办事。坚持按原则办事、按制度办事、按程序办事，把依法行政贯穿到政府决策、执行和监督的全过程，严格依照法定权限和程序行使权力、履行职责。深入推进政务公开和政府信息公开，不断提高政府工作的透明度和公众参与度。自觉接受人大、政协、人民群众和新闻媒体的监督，认真办理人大代表建议和政协委员提案。廉洁从政，干净干事。自觉遵守廉政准则，严格落实“一岗双责”，常怀感恩之心，常怀敬畏之心，常怀律己之心，切实做到反腐倡廉常抓不懈、拒腐防变警钟长鸣，努力打造一支政治坚定、风清气正、人民满意的公务员队伍，以勤政廉政的作为取信于民。

　　各位代表，事业凝聚人心，行动铸就辉煌!让我们紧密团结在以习近平同志为总书记的党中央周围，在省委、省政府和市委的正确领导下，凝心聚力，锐意进取，勇于开拓，攻坚克难，为建设富裕、秀美、宜居、和谐鹰潭而不懈奋斗！
